# Lenalidomide versus bortezomib maintenance after frontline autologous stem cell transplantation for multiple myeloma

**DOI:** 10.1038/s41408-020-00390-3

**Published:** 2021-01-07

**Authors:** Marc-Andrea Baertsch, Elias K. Mai, Thomas Hielscher, Uta Bertsch, Hans J. Salwender, Markus Munder, Stephan Fuhrmann, Ulrich Dührsen, Peter Brossart, Kai Neben, Jana Schlenzka, Christina Kunz, Marc S. Raab, Jens Hillengaß, Anna Jauch, Anja Seckinger, Dirk Hose, Steffen Luntz, Pieter Sonneveld, Henk Lokhorst, Hans Martin, Martin Goerner, Martin Hoffmann, Hans-Walter Lindemann, Helga Bernhard, Igor W. Blau, Christof Scheid, Britta Besemer, Katja C. Weisel, Mathias Hänel, Jan Dürig, Hartmut Goldschmidt, German-Speaking Myeloma Multicenter Group (GMMG)

**Affiliations:** 1 Department of Internal Medicine V, University Hospital Heidelberg, Heidelberg, Germany; 2 Division of Biostatistics, German Cancer Research Center (DKFZ), Heidelberg, Germany; 3 Nationales Centrum für Tumorerkrankungen (NCT) Heidelberg, Heidelberg, Germany; 4 Asklepios Tumorzentrum Hamburg, AK Altona and AK St. Georg, Hamburg, Germany; 5 Department of Internal Medicine III, University Medical Center Mainz, Mainz, Germany; 6 Department of Hematology and Stem Cell Transplantation, Helios Hospital Berlin Buch, Berlin, Germany; 7 Department of Hematology, University Clinic Essen, Essen, Germany; 8 University Hospital Bonn, Bonn, Germany; 9 Department of Hematology and Oncology, Klinikum Baden Baden, Baden Baden, Germany; 10 Institute of Child Nutrition, Max Rubner Institute, Federal Research Institute of Nutrition and Food, Karlsruhe, Germany; 11 Roswell Park Comprehensive Cancer Center, Buffalo, NY USA; 12 Institute of Human Genetics, University of Heidelberg, Heidelberg, Germany; 13 Department of Hematology and Immunology, Myeloma Center Brussels, Jette, Belgium; 14 Coordination Centre for Clinical Trials (KKS) Heidelberg, Heidelberg, Germany; 15 Department of Hematology, Erasmus MC Cancer Institute, Rotterdam, the Netherlands; 16 Department of Hematology, VU University Medical Center, Amsterdam, the Netherlands; 17 Department of Medicine, Hematology/Oncology, Goethe-University of Frankfurt, Frankfurt, Germany; 18 Department of Hematology, Oncology and Palliative Care, Klinikum Bielefeld, Bielefeld, Germany; 19 Medical Clinic A, Klinikum Ludwigshafen, Ludwigshafen, Germany; 20 Department of Hematology and Oncology, Katholisches Krankenhaus Hagen, Hagen, Germany; 21 Internal Medicine V, Klinikum Darmstadt, Darmstadt, Germany; 22 Medical Clinic, Charité University Medicine Berlin, Berlin, Germany; 23 Department of Internal Medicine I, University Hospital Köln, Köln, Germany; 24 Department of Hematology, Oncology and Immunology, University Hospital Tübingen, Tübingen, Germany; 25 Department of Oncology, Hematology and Bone Marrow Transplantation with Section of Pneumology, University Medical Center Hamburg-Eppendorf, Hamburg, Germany; 26 Department of Internal Medicine III, Klinikum Chemnitz, Chemnitz, Germany

**Keywords:** Myeloma, Medical research

## Abstract

Lenalidomide (LEN) maintenance (MT) post autologous stem cell transplantation (ASCT) is standard of care in newly diagnosed multiple myeloma (MM) but has not been compared to other agents in clinical trials. We retrospectively compared bortezomib (BTZ; *n* = 138) or LEN (*n* = 183) MT from two subsequent GMMG phase III trials. All patients received three cycles of BTZ-based triplet induction and post-ASCT MT. BTZ MT (1.3 mg/m^2^ i.v.) was administered every 2 weeks for 2 years. LEN MT included two consolidation cycles (25 mg p.o., days 1–21 of 28 day cycles) followed by 10–15 mg/day for 2 years. The BTZ cohort more frequently received tandem ASCT (91% vs. 33%) due to different tandem ASCT strategies. In the LEN and BTZ cohort, 43% and 46% of patients completed 2 years of MT as intended (*p* = 0.57). Progression-free survival (PFS; HR = 0.83, *p* = 0.18) and overall survival (OS; HR = 0.70, *p* = 0.15) did not differ significantly with LEN vs. BTZ MT. Patients with <nCR after first ASCT were assigned tandem ASCT in both trials. In patients with <nCR and tandem ASCT (LEN: *n* = 54 vs. BTZ: *n* = 84), LEN MT significantly improved PFS (HR = 0.61, *p* = 0.04) but not OS (HR = 0.46, *p* = 0.09). In conclusion, the significant PFS benefit after eliminating the impact of different tandem ASCT rates supports the current standard of LEN MT after ASCT.

## Introduction

High dose chemotherapy (HDCT) followed by autologous stem cell transplantation (ASCT) substantially improves PFS in newly diagnosed MM and remains a standard of care for fit patients in the era of novel agents^1–4^. Immunomodulatory drugs (IMiDs), proteasome inhibitors (PIs), monoclonal antibodies and their combinations are now incorporated into the various treatment sequences of MM based on improved PFS and OS^5–8^. The favorable toxicity profiles of the novel agents have spurred development of continuous treatment regimens which can be administered until disease progression and further improve survival^8,9^.

LEN maintenance treatment (MT) after HDCT/ASCT has become standard of care based on three phase III clinical trials (CALGB 100104^10^, GIMEMA RV-MM-PI-209^2^, IFM 2005-02^11^) documenting superior progression-free survival (PFS) and a large meta-analysis (*n* = 1208) revealing an estimated overall survival (OS) benefit of 2.5 years versus observation or placebo^8^. Results from the MRC XI trial recently confirmed the PFS benefit^12^.

However, about 30% of patients discontinue LEN MT due to a diverse range of treatment emergent adverse events^8^. These include hematotoxicity, general disorders, diarrhea, second primary malignancies (SPM) and others. Many clinical trials have investigated alternative agents for MT ranging from glucocorticoids, interferon alpha and thalidomide to bortezomib (BTZ) and—most recently—ixazomib^13^. While a PFS benefit and in some cases an OS benefit has been reported for these regimens, comparative clinical trials against today’s standard of LEN MT have not been published. Several institutions have retrospectively reported their experiences with different agents for MT in clinical practice, but interpretability of these analyses is limited by selection of MT according to baseline patient factors including cytogenetic risk, which makes direct comparison of the different MT agents difficult^14,15^.

BTZ is frequently recommended as an alternative, risk-adapted approach for MT in patients with adverse disease characteristics, e.g., by MAYO clinic SMART criteria^16^ and German-speaking Myeloma Multicenter Group (GMMG) standards^5,17,18^. The randomized phase III HOVON-65/GMMG-HD4 trial^5,17–19^ reported amelioration of the poor prognosis associated with deletion 17p and renal insufficiency with a regimen incorporating BTZ into induction and MT vs. classical chemotherapy induction and thalidomide MT.

Data regarding the benefit of LEN MT in patients with adverse disease characteristics is somewhat conflicting. The meta-analysis by McCarthy et al.^8^ showed only a marginal effect of LEN maintenance versus placebo/observation on PFS in the high-risk subgroups of patients with elevated LDH, impaired renal function or adverse cytogenetics such as deletion 17p. No OS benefit was observed in these subgroups and additionally in patients with international staging system (ISS) stage III disease. Conversely, in the MRC XI trial^20^ the PFS benefit of LEN maintenance versus placebo was consistent across subgroups including patients with (ultra) high-risk cytogenetics and ISS stage III. OS was significantly improved by LEN MT in the transplant-eligible part of the MRC XI trial and no heterogeneity between cytogenetic risk groups and ISS stages was observed.

To the best of our knowledge no prospective clinical trial has compared BTZ with LEN MT in the post-ASCT setting yet. We therefore sought to exploit the similar designs of the subsequently conducted, multicenter phase III trials GMMG-HD4^5,19^ and MM5^21,22^ to compare BTZ and LEN MT and investigate treatment effects in relevant disease subgroups without the bias of risk-adapted MT choice.

## Methods

### Trials analyzed

The present analysis retrospectively compared MT regimens in newly-diagnosed, transplant-eligible MM from two subsequently conducted multicenter phase III trials: GMMG-HD4, the German part of the HOVON-65/GMMG-HD4 trial (enrollment period: 05/2005-05/2008, EudraCT No. 2004-000944-26), and GMMG-MM5 (enrollment period: 07/2010-11/2013, EudraCT No. 2010-019173-16). Primary endpoints of both trials have been published previously^19,21,22^. For details and study protocols please see the original publications. The trials were conducted in accordance with the European Clinical Trial Directive, the Declaration of Helsinki and local ethics committees. All patients gave written informed consent. All authors had access to the primary clinical trial data.

### Study cohorts and treatment

The analysis included 138 and 183 patients prior to start of maintenance (HD4, study arm B) and consolidation (MM5, study arms A1/A2) therapy, respectively. All patients included in the present analysis received three cycles of a PI-based induction therapy: either PAD/PAd (BTZ/doxorubicin with either high- [HD4 arm B] or low-dose [MM5 arm A1] dexamethasone, *n* = 138/88)^23^ or VCD (BTZ/cyclophosphamide/dexamethasone; MM5 arm A2, *n* = 95). In the HD4 trial, patients were intended to receive upfront tandem high dose melphalan (200 mg/m^2^, HDM) and ASCT followed by BTZ MT (1,3 mg/m^2^ i.v. every 2 weeks) for 2 years (HD4 study arm B). Patients in the MM5 trial received a single HDM/ASCT, and only in case of less than near complete response (<nCR) after first HDM/ASCT a tandem HDM/ASCT was conducted. Thereafter, LEN MT was administered and included two consolidation cycles (25 mg p.o., days 1–21, repeated on day 29, for 2 cycles) followed by 2 years of initially 10 mg/day p.o., which was increased up to 15 mg/day after 3 months in case of good tolerability. Due to the upper age limit of 65 years in the HD4 trial, patients from the MM5 trial older than 65 years were excluded from the present analysis.

### Study endpoints and assessments

All analyses in the present study are exploratory and retrospective, comparing individual patient data from two separate trials. Endpoints were PFS and OS, OS from first relapse/progression, time on MT and toxicities during MT. Subgroup analyses of PFS and OS according to baseline parameters (cytogenetics, ISS, LDH and renal function) and tandem HDM/ASCT in patients with <nCR after first HDM/ASCT were conducted.

PFS and OS events were recorded from start of BTZ MT [HD4] or LEN consolidation therapy [MM5]. PFS events included relapse from CR, progressive disease (PD) or death from any cause, whichever occurred first. Patients with PD after induction were allowed to stay on protocol treatment in the absence of new or progressive end organ damage. These patients are excluded for PFS analysis but evaluated for OS. To harmonize follow-up of the two trials, administrative censoring at 60 months was performed.

Eligibility criteria^19,22^, sample processing for fluorescence in-situ hybridization (FISH)^17^ and response assessments according to the International Myeloma Working Group (IMWG) criteria^24^ have been described previously. Near CR (nCR) as a subcategory of very good partial response (VGPR) was defined as the absence of serum and urine M-protein on electrophoresis and/or standard 24-hour urinary measurement with a positive or missing immunofixation status in the serum and/or urine and/or missing bone marrow examination. High-risk (HR) cytogenetics were defined as either deletion del17p13 and/or translocation *t*(4;14) and/or gain 1q21 > 3 copies; standard risk (SR) cytogenetics were defined as absence of HR cytogenetics. Adverse events (AEs) were recorded applying the NCI CTCAE criteria (only if ≥ °3 for the present analysis; version 3.0 [HD4] and 4.0 [MM5]). Infections were retrospectively categorized according to the suspected infectious agent (bacterial, viral, unknown). Serious adverse events (SAE) were recorded independent from the CTCAE grade.

### Statistical analyses

Fisher’s exact test and Wilcoxon test were used to compare categorical and continuous parameters between groups. Distribution of PFS and OS times was estimated by the method of Kaplan and Meier. Univariable and multivariable Cox regression was used to estimate the treatment effect. Hazard ratio (HR) gives increase in risk for LEN compared to BTZ, i.e., a HR > 1 means BTZ is beneficial whereas a HR < 1 means LEN is beneficial. Likelihood-ratio test of the model with and without interaction term between treatment and risk factor was performed to determine treatment subgroup effects. In multivariable Cox models, missing covariate values were multiply imputed using the mice algorithm^25^. All statistical analyses were done with R 3.5 (www.r-project.org).

## Results

BTZ and LEN cohorts were balanced regarding baseline characteristics at the time of initiation of induction treatment (Table 1). Median age was 57.0 years (31.0–65.0) and 94% of patients had a WHO performance status of 0/1. Adverse prognostic features were present in 20% according to ISS (ISS III), in 24% according to HR cytogenetics, in 17% according to elevated lactate dehydrogenase (LDH) levels and in 9% according to renal impairment (RI).

**Table 1:** Baseline characteristics at the time of treatment initiation, i.e., before bortezomib-based induction treatment.

	BTZ cohort (*n* = 138)	LEN cohort (*n* = 183)	*p*
Age (median)	56 (50–61)	57 (52–61)	0.22
Sex			
Female	52 (38%)	80 (44%)	0.30
Male	86 (62%)	103 (56%)	
WHO PS			
0	66 (48%)	87 (48%)	1.00
1	64 (46%)	84 (46%)	
>1	8 (6%)	11 (6%)	
MM isotype			
Heavy chain			
IgG	77 (56%)	112 (61%)	0.45
IgA	35 (25%)	34 (19%)	
IgD	1 (1%)	2 (1%)	
LCD	24 (17%)	35 (19%)	
Light chain			
Kappa	90 (66%)	126 (69%)	0.63
Lambda	47 (34%)	57 (31%)	
ISS			
I	49 (38%)	85 (47%)	0.33
II	52 (40%)	63 (34%)	
III	28 (22%)	35 (19%)	
Cytogenetics			
del17p13	14 (12%)	17 (10%)	0.70
* t*(4;14)	14 (12%)	14 (9%)	0.43
gain1q > 3 copies	8 (7%)	13 (8%)	0.66
HR^a^	28 (24%)	37 (24%)	1.00
SR^b^	89 (76%)	116 (76%)	
Elevated LDH	22 (17%)	32 (18%)	0.88
Renal impairment^c^	17 (12%)	13 (7%)	0.12

*BTZ* bortezomib, *ISS* international staging system, *LDH* lactate dehydrogenase, *LCD* light chain disease, *LEN* lenalidomide, *MM* multiple myeloma, *WHO PS* world health organization performance status.

^a^HR (high risk) cytogenetics were defined as presence of del(17p13) and/or *t*(4;14) and/or gain1q21 > 3 copies.

^b^SR (standard risk) cytogenetics were defined as absence of high risk features.

^c^Serum creatinine >2 mg/dl. Data are median (interquartile range) or *n* (%).

The rate of tandem ASCT was significantly lower in the LEN cohort (LEN: 33% vs. BTZ: 91%; *p*  < 0.001) and the proportion of patients achieving at least nCR after completion of the HDM/ASCT phase (single or tandem) were higher in the LEN cohort (LEN: 52% vs. BTZ: 39%, *p*  = 0.02) but ≥VGPR did not differ significantly (LEN: 71% vs. BTZ: 62%;. *p*  = 0.11; Table 2). The median interval from initiation of induction treatment to initiation of MT was 8.5 months (interquartile range [IQR] 7.7–10.3) and 9.6 months (IQR 8.6–11.0) in the LEN and BTZ cohorts, respectively (*p*  = 0.0001). Median time on MT in the LEN cohort was 24.0 months (IQR 10.6–25.9, including two cycles of LEN consolidation) versus 21.5 months (IQR 12.7–24.1) in the BTZ cohort (*p*  = 0.0017). Rates of any documented dose modification (including dose reductions, delays, interruptions and resumptions, and discontinuations) were similar between the two MT strategies (LEN: 141/183 [77%] vs. 102/138 [74%]; *p*  = 0.60). The fraction of patients completing two years of MT according to trial protocols was comparable in both cohorts (LEN: 78/183 [43%] vs. BTZ: 64/138 [46%]; *p*  = 0.57).

**Table 2:** Response rates prior to start of maintenance therapy.

	BTZ cohort *n* = 134	LEN cohort *n* = 179	*p*
Single categories			<0.001
CR	41 (31%)	38 (21%)	
nCR	11 (8%)	55 (31%)	
VGPR	31 (23%)	34 (19%)	
PR	50 (37%)	42 (23%)	
MR	1 (1%)	9 (5%)	
SD	0 (0%)	1 (1%)	
Combined categories			
CR/nCR	52 (39%)	93 (52%)	0.02
≥VGPR	83 (62%)	127 (71%)	0.11
PR/MR/SD	51 (38%)	52 (29%)	0.11

Data are *n* (%).

*CR* complete response, *nCR* near CR, *VGPR* very good partial response, *PR* partial response, *MR* minimal response, *SD* stable disease.

**Fig. 1:**
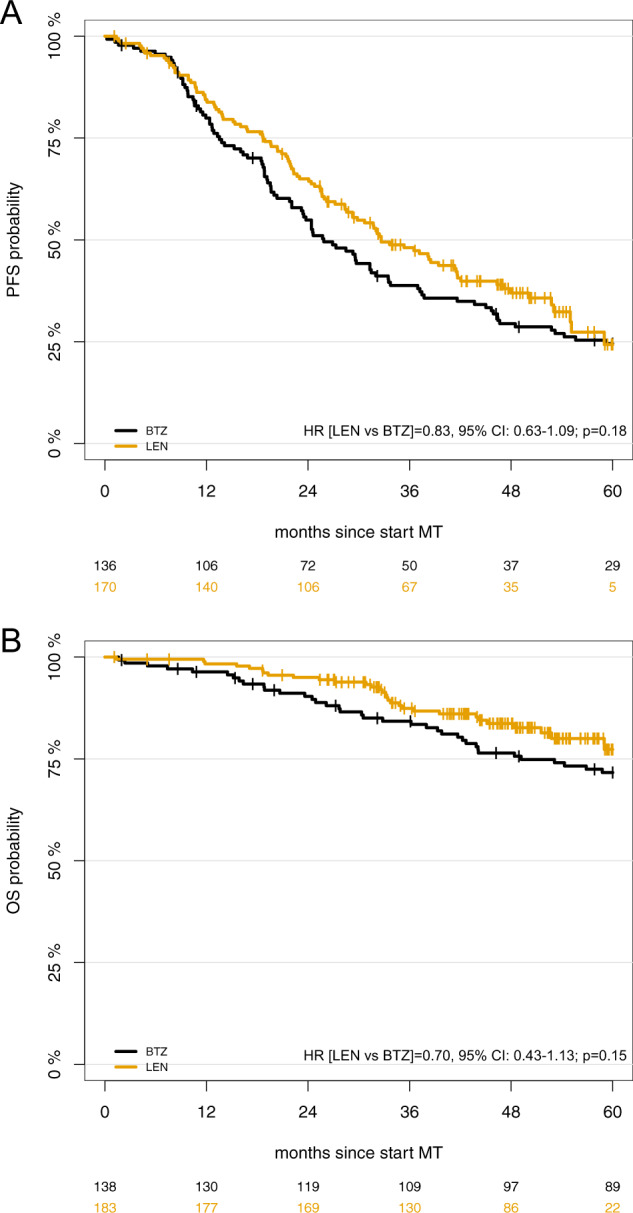
Progression-free (PFS) and overall survival (OS) in the overall cohorts. Kaplan–Meier curves are shown for (**a**) PFS and (**b**) OS. LEN lenalidomide, BTZ bortezomib.

After a median follow up of 49 and 60 months, 104 (57%) and 99 (65%) PFS events have occurred in the LEN and BTZ cohorts, respectively. Median PFS from initiation of MT was not significantly different in the LEN and BTZ cohorts (32.7 vs. 25.9 months; HR [LEN vs BTZ] = 0.83, 95% confidence interval [95% CI]: 0.63–1.09; *p*  = 0.18; Fig. 1a). Thirty and 37 OS events have occurred. Median OS from initiation of MT was not reached in either cohort; OS was not significantly different between cohorts (HR [LEN vs BTZ] = 0.70, 95% CI: 0.43–1.13; *p*  = 0.15; Fig. 1b). OS at 4 years was 84% and 76% in the LEN and BTZ cohort, respectively. OS from first relapse did not differ significantly between the two cohorts (HR = 0.73; 95% CI 0.42–1.24; *p*  = 0.25; Supplementary Fig. 1).

Multivariate analyses considering established baseline prognostic factors and response status after completion of the transplant phase (Table 3) were calculated. Effect size of MT (LEN vs. BTZ) on PFS (HR = 0.83; *p*  = 0.20) and OS (HR 0.65; *p*  = 0.11) were comparable with univariate analyses. Elevated LDH and HR cytogenetics were prognostic for both PFS and OS, while the ISS stages II and III were prognostic for OS.

**Table 3:** Multivariate analysis on PFS and OS.

	PFS		OS	
	HR (95% CI)	*p*	HR (95% CI)	*p*
MT (LEN vs. BTZ)	0.83 (0.62–1.10)	0.20	0.65 (0.39–1.10)	0.11
ISS (II vs. I)	1.29 (0.93–1.79)	0.13	2.12 (1.13–3.99)	0.02
ISS (III vs. I)	1.20 (0.76–1.92)	0.44	2.25 (1.05–4.81)	0.04
Cytogenetics (high vs. standard risk)	1.55 (1.10–2.18)	0.01	2.10 (1.14–3.88)	0.02
LDH (elevated vs. normal)	1.63 (1.12–2.36)	0.01	3.31 (1.84–5.93)	<0.001
RI (yes vs. no)	0.87 (0.48–1.57)	0.64	0.62 (0.26–1.49)	0.29
Age (continuous)	1.01 (0.99–1.03)	0.47	1.03 (1.00–1.07)	0.08
Response^a^ (nCR/CR vs. <nCR)	0.79 (0.59–1.06)	0.12	0.86 (0.51–1.46)	0.58

Cox regression models including PFS: *n* = 306 patients, events *n* = 203; OS: *n* = 321 patients, events *n* = 67.

*BTZ* bortezomib, *CI* confidence interval, *CR* complete response, *HR* hazard ratio, *ISS* international staging system, *LDH* lactate dehydrogenase, *LEN* lenalidomide, *MT* maintenance treatment, *nCR* near complete response, *RI* renal impairment (serum creatinine >2 mg/dl).

^a^Response prior to start of consolidation / maintenance therapy

**Fig. 2:**
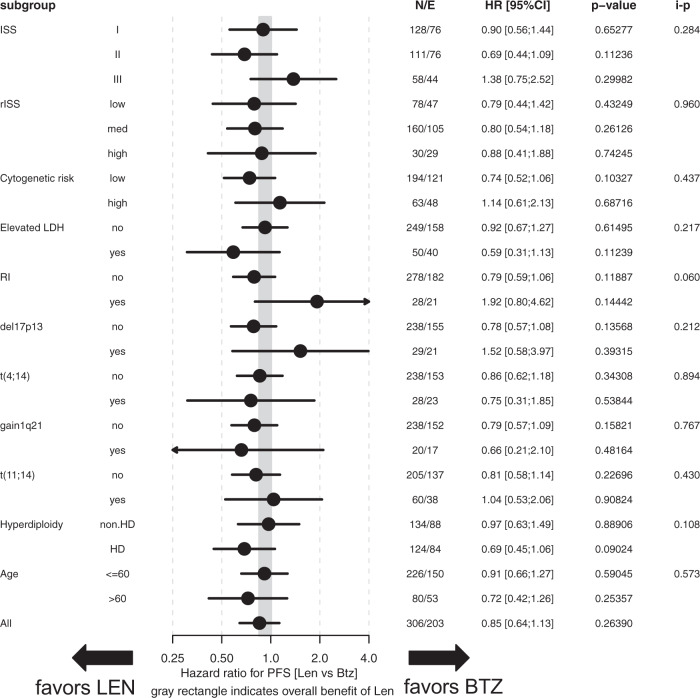
Subgroup analyses on progression-free survival (PFS) according to baseline factors. Multivariate subgroup analyses adjusted for response (CR/nCR vs.<nCR) were calculated for PFS in the overall cohort. ISS international staging system, rISS revised ISS, cytogenetic risk: high vs. standard [=low] risk, Elevated LDH (elevated [yes] vs. normal [no]), RI renal impairment, defined as serum creatinine (>2 mg/dl [yes] vs. <2 mg/dl [no]); Hyperdiploidy cytogenetic hyperdiploidy [HD].

**Fig. 3:**
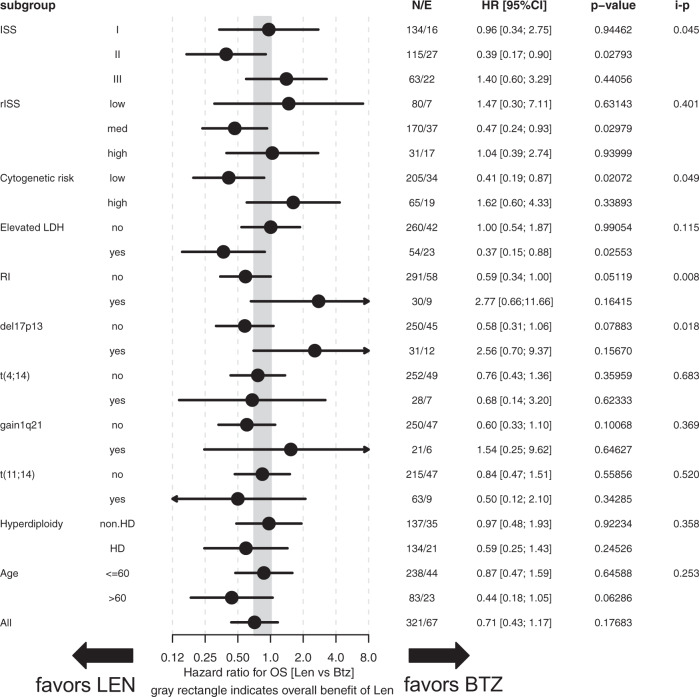
Subgroup analyses on overall survival (OS) according to baseline factors. Multivariate subgroup analyses adjusted for response (CR/nCR vs.<nCR) were calculated for OS in the overall cohort. ISS international staging system, rISS revised ISS, cytogenetic risk: high vs. standard [=low] risk; Elevated LDH: LDH (elevated [yes] vs. normal [no]); RI: renal impairment, defined as serum creatinine (>2 mg/dl [yes] vs. <2 mg/dl [no]); *Hyperdiploidy:* cytogenetic hyperdiploidy [HD].

Multivariate subgroup analyses adjusted for response status after completion of the transplant phase/prior to start of MT (CR/nCR vs.<nCR) revealed no significant differential treatment effects for LEN vs. BTZ regarding PFS (Fig. 2). Response-adjusted analyses of OS (Fig. 3) revealed significant differential treatment effects (LEN vs. BTZ) for subgroups according to cytogenetics (SR vs. HR; interaction p [i-p]=0.049), deletion17p (i-p = 0.018), RI (i-p = 0.008) and ISS (i-p = 0.045). No benefit from LEN vs. BTZ MT was observed in patients with deletion17p (HR = 2.56; 95% CI: 0.7–9.37; *p*  = 0.16) or RI (HR = 2.77; 95% CI: 0.66–11.66; *p*  = 0.16). Univariate subgroup analyses on PFS and OS were similar to response adjusted, multivariate analyses (Supplementary Figs. 2 and 3).

In addition, when patients with RI or deletion 17p were excluded from the overall analyses, the benefit of LEN vs. BTZ MT became significant in the remaining patients both for PFS (HR = 0.7, 95% CI: 0.5–0.97, *p* = 0.03) and OS (HR = 0.46, 95% CI: 0.24–0.87, *p* = 0.02). Differential treatment effects according to cytogenetics (SR vs. HR; i-p = 0.92) and ISS (i-p = 0.52) also disappeared when patients with deletion 17 p or RI were excluded.

**Fig. 4:**
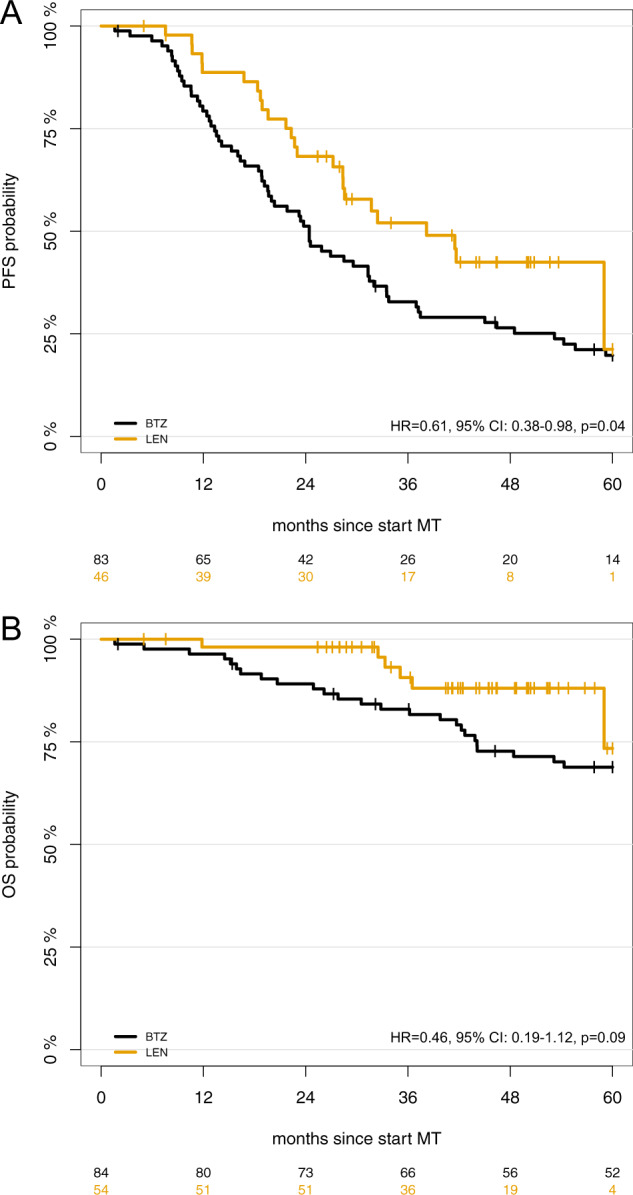
Progression-free (PFS) and overall survival (OS) in patients receiving tandem autologous stem cell transplants (ASCT) for <nCR after first ASCT. Kaplan–Meier curves are shown for (**a**) PFS and (**b**) OS. LEN lenalidomide, BTZ bortezomib.

To account for differences in tandem ASCT rates, a subgroup analysis was conducted including only patients with suboptimal response (<nCR) after first ASCT and thus receiving a tandem ASCT in both trials. In these *n* = 54 (LEN cohort) and *n* = 84 (BTZ cohort) patients, LEN MT was associated with significantly superior PFS (HR = 0.61, 95% CI: 0.38–0.98, *p*  = 0.04; Fig. 4a) but not OS (HR = 0.46, 95% CI, 0.19–1.12, *p*  = 0.09; Fig. 4b).

Grade ≥3 AE were reported in 123 (67%) and 79 (57%) (*p*  = 0.08) patients in the LEN and BTZ cohort, respectively (Table 4). Grade ≥3 leukopenia/neutropenia was more frequent in the LEN cohort (79/183 [43%] vs. 3/138 [2%]; *p*  < 0.0001). However, this did not translate into an increase in grade ≥3 infections in the LEN cohort (40/183 [22%] vs. 42/138 [30%]; *p*  = 0.09). Grade ≥3 viral infections, especially varicella zoster virus (VZV) reactivations (14/138 [10%] vs. 7/183 [4%]; *p*  = 0.04) were more frequent in the BTZ cohort. More grade ≥3 peripheral neuropathies (PNP) were reported in the BTZ cohort (12/138 [9%] vs. 3/183 [2%]; *p*  = 0.01).

**Table 4:** Grade ≥3 adverse events during maintenance treatment.

	BTZ cohort *n* = 138	LEN cohort *n* = 183	*p*
At least one AE grade ≥3	79 (57%)	123 (67%)	0.08
Neutropenia/Leukocytopenia	3 (2%)	79 (43%)	<0.0001
Anemia	–	3 (2%)	0.26
Thrombocytopenia	6 (4%)	19 (10%)	0.06
Infections	42 (30%)	40 (22%)	0.09
* Bacterial infections (susp.)*	15 (11%)	21 (12%)	1.00
* Viral infections (susp.)*	23 (17%)	14 (8%)	0.01
* VZV*	14 (10%)	7 (4%)	0.04
* Unknown etiology*	9 (7%)	11 (6%)	1.00
Gastrointestinal disorders	7 (5%)	7 (4%)	0.59
Peripheral neuropathy	12 (9%)	3 (2%)	0.01
DVT/PE	2 (1%)	4 (2%)	0.70
Rash	2 (1%)	7 (4%)	0.31

Data are *n* (%). Only events ≥ CTCAE grade 3 are analysed.

*DVT/PE* Deep vein thrombosis/pulmonary embolism, *VZV* Varicella zoster virus, *LEN* lenalidomide, BTZ bortezomib.

## Discussion

This is the first comparative report of LEN and BTZ for MT after upfront HDM/ASCT for newly diagnosed MM without the bias of risk-adapted MT choice. While survival with LEN vs. BTZ MT did not differ significantly in the overall cohorts, a significant PFS benefit for LEN MT was observed after eliminating the impact of different tandem ASCT rates.

The strength of our analysis lies in the risk-independent use of both BTZ and LEN MT in the two consecutive phase III clinical trials GMMG-HD4 and -MM5. The similar design of these two multicenter trials with three cycles of BTZ-based triplet induction therapy followed by HDM/ASCT and MT allowed for comparison of the two MT strategies using high-quality, patient-level data. Due to similar eligibility criteria in both trials, baseline characteristics of our BTZ and LEN MT cohorts at the time of treatment initiation including established prognostic factors such as the ISS and HR cytogenetics were well balanced. Furthermore, the two BTZ-based induction regimens used in our patients have been shown to produce equivalent overall response rates, rates of deep responses (≥VGPR) and PFS/OS^21,23^.

A limitation of our analysis—apart from its retrospective nature—are the different tandem ASCT policies in the two trials. The general recommendation of tandem ASCT for all patients in the HD4 trial versus response-adapted tandem ASCT for patients in the MM5 trial resulted in a significantly higher rate of tandem ASCT in the HD4/BTZ cohort. The effect of tandem ASCT on survival of patients with MM is still a matter of debate. In the pre-novel agent era two randomized phase III trials^26,27^ reported a benefit for tandem ASCT while long-term results of the randomized phase III trial GMMG-HD2^28^ performed in our study group showed no survival differences between single and tandem ASCT after a median follow-up of more than eleven years. In the novel agent era, two phase III clinical trials (EMN02^29^, STAMINA^30^) comparing single vs. tandem ASCT also yielded conflicting results. In studies reporting a benefit, patients with suboptimal response after the first ASCT were amongst those deriving the greatest benefit from a tandem ASCT^26,27^. These patients, i.e., patients with <nCR were assigned a tandem transplant in both the LEN and the BTZ cohort. Still, the lower rate of tandem ASCT in the LEN MT cohort may explain the absence of a significant survival benefit for LEN MT in the overall cohorts. In an attempt to eliminate the impact of different tandem ASCT rates, patients who received tandem ASCT for suboptimal response in both trials were compared. In this subgroup the PFS benefit with LEN MT was statistically significant.

An important aspect to consider in interpreting our results is that MM5/LEN MT after BTZ-based induction treatment constitutes a class switch from PI to IMiD, whereas patients in the HD4/BTZ cohort never received an IMiD during their frontline treatment. With the increasingly used BTZ/LEN/dexamethasone (VRD), BTZ/thalidomide/dexamethasone (VTD) and carfilzomib/LEN/dexamethasone (KRD) induction and consolidation regimens patients receiving MT are now frequently pre-exposed to both a PI and an IMiD which may impact on the relative efficacy of MT with a PI or IMiD. This aspect seems even more relevant since LEN MT in our cohort included two cycles of full dose LEN (25 mg/day), followed by 2 years of 10 mg/day (and up to 15 mg/day if tolerated), whereas BTZ was administered at a dose of 1.3 mg/m^2^ every two weeks for 2 years. Measured against the standard doses of the well-established LEN/dexamethasone (Rd; 25 mg/day, day 1–21 of 28 day cycles) and BTZ/dexamethasone (1.3 mg/m^2^, day 1, 4, 8, 11 of 21 day cycles) regimens, the LEN dose administered for MT was considerably higher than the BTZ dose.

Both MT regimens were well tolerated. Expectedly, hematological toxicity of LEN MT was more pronounced. This did not translate into an increase in infections in the LEN cohort. Slightly more infections - mainly viral infections - were observed in the BTZ cohort. In line with the established safety profile of BTZ, significantly more PNP were observed in the BTZ cohort. Intravenous administration of BTZ was standard of care at the time the HD4 trial was conducted, but subcutaneous administration of BTZ is now well established due to less AE, especially PNP^31,32^. Another PI - ixazomib (IXA) - causing less PNP (<1% grade ≥3) has been studied for MT. A recent phase III clinical trial (TOURMALINE-MM03) demonstrated superior PFS with IXA MT vs. placebo^33^. Oral bioavailability and good tolerability underscore the potential of IXA as MT but no comparative data with other MT agents is currently published.

An important issue with multiple available agents for MT is treatment stratification. The HRs of 0.83 and 0.70 for PFS and OS favoring the LEN vs. BTZ cohort and the significant PFS benefit of LEN MT in patients receiving tandem ASCT for suboptimal response support the current standard of LEN MT. An aim of our analysis was to reassess the previously reported benefit of BTZ MT in patients with RI or deletion 17p: The HD4 trial showed marked improvement of PFS and OS with BTZ-based induction and MT versus now obsolete vincristine/doxorubicine/dexamethasone (VAD) induction and thalidomide MT in these high-risk subgroups^5,17,18^. Using the original data from the HD4 trial we did not observe a significant benefit when compared against BTZ-based induction and LEN MT. However, we still found HR in the range of 1.5 to 2.8 in favor of BTZ MT both for PFS and OS pointing towards limited statistical power.

While our results need to be interpreted with caution due to the retrospective nature and the limitations discussed above, they support the current standard use of LEN MT. In patients that cannot tolerate LEN MT, BTZ may be considered as an alternative. Randomized controlled trials comparing different MT agents and combinations are warranted, especially in patients with high-risk features such as deletion 17p and RI.

## Supplementary information

Supplement - GMMG trial sites

Supplementary Figure 1

Supplementary Figure 2

Supplementary Figure 3
