# Supplementary material for: Lenalidomide versus bortezomib maintenance after frontline autologous stem cell transplantation for multiple myeloma
Source: Blood Cancer J. 2021 Jan 7;11(1):1. doi: 10.1038/s41408-020-00390-3 (PMC7791127; doi:10.1038/s41408-020-00390-3)
Supplement: Supplementary file 1 — Supplement - GMMG trial sites [file 41408_2020_390_MOESM1_ESM.doc]

**Involved German-Speaking Myeloma Multicenter Group (GMMG) trial sites**

| **GMMG HD4 trial** | **GMMG MM5 trial** |
| --- | --- |
| Medizinische Klinik IV  Hämatologie und Onkologie  Klinikum Bayreuth GmbH  Bayreuth  III. Medizinische Abteilung (Hämatologie/Onkologie)  Charité, Campus Benjamin Franklin  Berlin  Klinikum Bielefeld  Klinik für Hämatologie  Bielefeld  Medizinische Universitätsklinik Knappschaftskrankenhaus  Bochum  Studienzentrum Paul-Ehrlich-Haus  Bonn  Klinikum Chemnitz GmbH  Innere Medizin III  Chemnitz  Carl-Thiem-Klinikum Cottbus  II. Med. Klinik  Cottbus  Städtisches Klinikum Dessau  Klinik für Innere Medizin  Dessau  Universitätsklinikum Essen  Klinik für Hämatologie  Essen  Klinikum Essen Süd  Ev. Krankenhaus Essen-Werden  Essen  Med. Klinik III  Klinikum d. J.-W.-Goethe-Universität  Frankfurt  Praxis für Hämatologie/Intern. Onkologie/Klinische Immunologie  Frankfurt/Main  Städtische Kliniken Frankfurt a. M.-Höchst  Klinik für Innere Medizin. Abt. 3  Frankfurt a. M.  Kath. Krankenhaus Hagen gGmbH  Abt. Hämatologie/Onkologie  Hagen  Abt. Hämatologie/Onkologie  Medizinische Klinik  Ev. Krankenhaus Hamm  Hamm  Allg. Krankenhaus Altona  II. Med. Abteilung  Hamburg  Klinikum Region Hannover GmbH  Städt. Krankenhaus Siloah  Med. Klinik III  Hannover  Univ.-Klinikum Heidelberg  Med. Klinik V  Heidelberg  Innere Medizin I  Universitätsklinikum Saarland  66421 Homburg/Saar  Klinikum Idar-Oberstein GmbH  Klinik für Knochenmarktransplantation  und Hämatologie/Onkologie GmbH  Idar-Oberstein  Medizinische Klinik I  Westpfalz-Klinikum GmbH  Kaiserslautern  Universitätsklinikum Köln  Klinik I Innere Medizin  Köln  Medizinische Klinik II  Klinikum Lippe-Lemgo  Lemgo  Klinikum der Stadt Ludwigshafen  am Rhein GmbH  Ludwigshafen a. Rh.  Städt. Krankenhaus Süd  Abt. Hämatologie/Onkologie  Lübeck  Universitätsklinikum Marburg und Giessen GmbH  Standort Marburg  Klinik für Hämatologie, Onkologie und Immunologie  Marburg  Johannes Wesling Klinikum Minden  Zentrum für Innere Medizin  Minden/Westf.  Krankenhaus Maria Hilf GmbH  Franziskuskrankenhaus  Med. Klinik I  Mönchengladbach  Krankenhaus München-Schwabing  1. Med. Abteilung  München  Städtisches Klinikum München GmbH  Klinikum Harlaching  München  Paracelsus Klinik  Zentrum für Tumordiagnostik u.-therapie  Hämatologie/Int. Onkologie  Osnabrück  St. Marien-Krankenhaus Siegen  Abteilung Hämatologie/Onkologie  Siegen  Evang. Diakoniewerk Schwäbisch Hall e.V.  Diakonie-Krankenhaus  Innere Abteilung  Schwäbisch Hall  Robert-Bosch-Krankenhaus  Zentrum für Innere Medizin  Stuttgart  Med. Klinik Universität Tübingen  Abtl. II/Hämatologie, Onkologie, Immunologie und Rheumatologie  Tübingen | Facharztpraxis Hämatologie und Onkologie  Aschaffenburg  Praxisnetzwerk Hämatologie/ Internistische Onkologie  Bad Honnef  Facharztpraxis Onkologie  Bad Kreuznach  Caritas Krankenhaus Bad Mergentheim  Medizinische Klinik 2  Bad Mergentheim  MVZ Baden-Baden  Medizinisches Versorgungszentrum des Klinikums Mittelbaden  Baden-Baden  Charité Universitätsmedizin Berlin  Campus Benjamin Franklin  III. Medizinische Abteilung  Berlin  Charité Universitätsmedizin Berlin  Charité Campus Mitte  Medizinische Klinik mit Schwerpunkt Onkologie und Hämatologie  Berlin  HELIOS Klinikum Berlin-Buch  Klinik für Hämatologie, Onkologie und Immunologie  Berlin  Medizinisches Versorgungszentrum (MVZ)  Onkol. Schwerpunkt am Oskar-Helene-Heim  Berlin  Facharztpraxis Onkologie, Gastroenterologie, Hämatologie, Palliativmedizin  Berlin  Onkologie Seestrasse  Berlin  Klinikum Bielefeld Mitte  Klinik für Hämatologie, Onkologie und Palliativmedizin  Bielefeld  Facharztpraxis Onkologie, Gastroenterologie, Hämostaseologie, Palliativmedizin  Bochum  Universitätsklinikum Bonn  Med. Klinik und Poliklinik III  Schwerpunkte Onkologie, Hämatologie und Rheumatologie  Bonn  Zaho – Bonn  Zentrum für ambulante Hämatologie und Onkologie  Bonn  Johanniter Krankenhaus Bonn  Internistische Onkologie  Bonn  Onkologie Rheinsieg  Praxisnetzwerk Hämatologie/Internistische Onkologie  Bonn-Beuel  Städt. Klinikum Braunschweig  Medizinische Klinik III  Braunschweig  Onkologische Schwerpunktpraxis  Braunschweig  Onkologische Praxis im Krankenhaus Buchholz  Buchholz  Onkologische Schwerpunktpraxis  Celle  Klinikum Chemnitz  Innere Medizin III  Chemnitz  Regiomed Kliniken GmbH  Medizinisches Versorgungszentrum (MVZ) Coburg des Klinikums Coburg  Coburg  Carl-Thiem-Klinikum Cottbus  II. Medizinische Klinik  Cottbus  Klinikum Darmstadt  Medizinische Klinik V Hämatologie/Onkologie  Darmstadt  Onkologische Schwerpunktpraxis  Darmstadt  Gemeinschaftspraxis für Hämatologie und Onkologie  Medizinisches Zentrum am St.-Josefs-Hospital  Dortmund  Fachpraxis für Hämatologie und Onkologie  Erfurt  Universitätsklinikum Essen  Klinik für Hämatologie  Essen  Evangelisches Krankenhaus Essen-Werden Zentrum für Innere Medizin  Klinik für Hämatologie, Onkologie und Stammzell-transplantation  Essen  St. Antonius-Hospital  Klinik für Hämatologie und Onkologie  Eschweiler  Universitätsklinikum Frankfurt  Goethe-Universität  Medizinische Klinik II  Hämatologie, Onkologie, Rheumatologie, Infektiologie  Frankfurt am Main  Agaplesion Medizinisches Versorgungszentrum (MVZ) Frankfurt  Frankfurt am Main  Krankenhaus Nordwest  Klinik für Onkologie und Hämatologie  Frankfurt am Main  Interdisziplinäres Facharztzentrum (IFS) Frankfurt  Ambulantes Krebszentrum (AKS)  Frankfurt am Main  Vitanus GmbH  Frankfurt am Main  Frankfurter Rotkreuz-Kliniken  Klinik Maingau  Abt. Hämatologie/Onkologie und Palliativmedizin  Frankfurt am Main  PIOH-Praxis für Internistische Onkologie und Hämatologie  Frechen  Onkologische Facharztpraxis  Gerlingen  Facharztpraxis für Hämatologie und Onkologie  Gießen  Kath. Krankenhaus Hagen  St.-Josefs-Hospital  Klinik für Hämatologie und Onkologie  Hagen  Asklepios Klinik Altona  Abteilung Onkologie mit Sektion Hämatologie  Hamburg  Asklepios Klinik St. Georg  Abteilung Hämatologie, Onkologie und Stammzelltransplantation  Hamburg  Hämatologisch-Onkologische Praxis Altona (HOPA)  Hamburg  Facharztpraxis für Hämatologie und Onkologie  Hamburg  OncoResearch Lerchenfeld UG  Hamburg  Evangelisches Krankenhaus Hamm gGmbH  Medizinische Klinik  Hämatologie / Onkologie  Hamm  Onkologische Schwerpunktpraxis  63450 Hanau  Klinikum Hanau GmbH  Medizinische Klinik III  63450 Hanau  Klinikum Region Hannover  Klinikum Siloah  Onkologie und Palliativmedizin  Hannover  Onkologisches Ambulanzzentrum OAZ Hannover  Hannover  Universitätsklinikum Heidelberg  Medizinische Klinik V  Heidelberg  Onkologische Schwerpunktpraxis Heidelberg  Onkologische Schwerpunktpraxis Heilbronn  Heilbronn  SLK Kliniken Heilbronn GmbH  Medizinische Klinik III  Heilbronn  Facharztpraxis für Hämatologie und Tumorerkrankungen  Henningsdorf  Universitätsklinikum des Saarlandes  Innere Medizin I  Homburg/Saar  Klinikum Idar-Oberstein  Medizinische Klinik I  Idar-Oberstein  Westpfalz-Klinikum  INN1  Kaiserslautern  Schwerpunktpraxis für Hämatologie und Onkologie  Kaiserslautern  Gemeinschaftspraxis für Hämatologie, Onkologie und Infektiologie  Karlsruhe  Institut für Versorgungsforschung in der Onkologie (InVo)  Koblenz  Universitätsklinikum Köln  Klinik I Innere Medizin  Köln  Onkologie Köln  Gemeinschaftspraxis für Onkologie und Hämatologie  Köln  Praxis Internistischer Onkologie und Hämatologie (PIOH)  Köln  Kliniken Köln, Krankenhaus Köln-Holweide  Köln  Facharztpraxis für Hämatologie, Onkologie und Gerinnung  Kronach  Onkologisches Zentrum Lebach  Caritaskrankenhaus Lebach  Lebach  Klinikum der Stadt Ludwigshafen  am Rhein  Medizinische Klinik A  Ludwigshafen a. Rh.  Onkologische Schwerpunktpraxis Lüneburg  Lüneburg  Universitätsmedizin der Johannes Gutenberg-Universität Mainz  III. Medizinische Klinik  Mainz  MED Facharztzentrum  Gemeinschaftspraxis für Hämatologie und Onkologie  Mainz  Universitätsmedizin Mannheim  III. Medizinische Klinik  Hämatologie und Internistische Onkologie  Mannheim  Mannheimer Onkologie Praxis  Mannheim  Facharztpraxis für Innere Medizin, Hämatologie und Onkologie  Mannheim  Praxis für Innere Medizin, Hämatologie und internistische Onkologie  Marburg  Mühlenkreiskliniken (AöR)  Johannes Wesling Klinikum Minden  Hämatologie/Onkologie, Hämostaseologie und Palliativmedizin  Minden  Kliniken Maria Hilf  Krankenhaus St. Franziskus  Klinik für Hämatologie, Onkologie und Gastroenterologie  Mönchengladbach  Städtisches Klinikum München  Klinikum Harlaching  Klinik für Hämatologie, Onkologie und Palliativmedizin  München  Facharztpraxis für Innere Medizin, Hämatologie und Onkologie  Neunkirchen  medius Kliniken  Klinik Nürtingen  Klinik für Innere Medizin  Onkologie, Hämatologie  Nürtingen  Onkologische Facharztpraxis  Oberhausen  Paracelsus Kliniken  Klinik Osnabrück  Innere Medizin / Hämatologie und Onkologie  Osnabrück  medius Kliniken  Klinik Ostfildern-Ruit  Innere Medizin, Gastroenterologie und Tumormedizin  Ostfildern  Medizinisches Versorgungszentrum am Siloah St. Trudbert-Klinikum  Pforzheim  Onkologische Praxis Pinneberg  Pinneberg  Gemeinschaftspraxis Innere Medizin/Onkologie  Pirmasens  Facharztpraxis für Onkologie  Rosenheim  Diakonie-Klinikum Schwäbisch Hall  Klinik für Innere Medizin III  Schwäbisch Hall  Zaho-Zentrum für ambulante Hämatologie und Onkologie  Siegburg  Diakonie Klinikum Jung-Stilling  Innere Medizin  Siegen  Gastroenterologie Onkologie Bodensee  Praxis Singen  Singen  Onkologische Schwerpunktpraxis Speyer  Speyer  Marienhospital Stuttgart  Zentrum für Innere Medizin III  Onkologie, Hämatologie, Palliativmedizin-  Stuttgart  Krankenhaus der Barmherzigen Brüder Trier  Innere Medizin I  Trier  Klinikum Mutterhaus der Borromäerinnen Trier  Innere Medizin I  Trier  Onkologische Schwerpunktpraxis am Brüderkrankenhaus  Trier  Praxis für Innere Medizin, Nephrologie, Hämatologie und Onkologie  Trier  Onkologie Rheinsieg  Praxisnetzwerk Hämatologie und Internistische Onkologie  Troisdorf  Universität Tübingen  Medizinische Klinik  Abtl. II/Hämatologie, Onkologie, Immunologie und Rheumatologie  Tübingen  OMM Optimed Mundial GmbH  Viersen  Facharztpraxis für Onkologie  Wendlingen/Esslingen  Ammerland Klinik  Medizinische Klinik  Westerstede  Facharztpraxis für Onkologie  Westerstede  Onkologische Schwerpunktpraxis  Wolfsburg – Helmstedt  Praxis Wolfsburg  Wolfsburg |
