# Supplementary material for: Lenalidomide versus bortezomib maintenance after frontline autologous stem cell transplantation for multiple myeloma
Source: Blood Cancer J. 2021 Jan 7;11(1):1. doi: 10.1038/s41408-020-00390-3 (PMC7791127; doi:10.1038/s41408-020-00390-3)
Supplement: Supplementary file 2 — Supplementary Figure 1 [file 41408_2020_390_MOESM2_ESM.pdf]

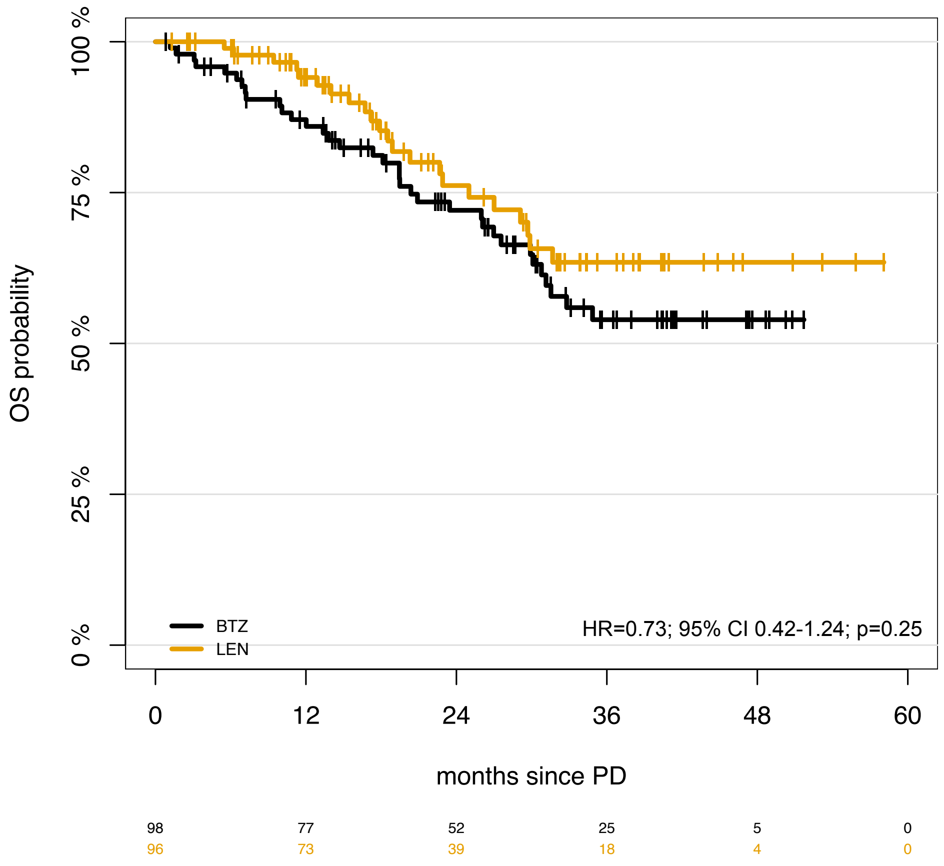

Supplemental figure 1: Overall survival (OS) from first relapse in the overall cohorts
Kaplan-Meier curves are shown for **(A)** PFS and **(B)** OS. *LEN:* lenalidomide; *BTZ:* bortezomib

Supplemental Figure 1
