# Supplementary material for: Lenalidomide versus bortezomib maintenance after frontline autologous stem cell transplantation for multiple myeloma
Source: Blood Cancer J. 2021 Jan 7;11(1):1. doi: 10.1038/s41408-020-00390-3 (PMC7791127; doi:10.1038/s41408-020-00390-3)
Supplement: Supplementary file 3 — Supplementary Figure 2 [file 41408_2020_390_MOESM3_ESM.pdf]

subgroup		N/E	HR [95%CI]	p–value	i-p
ISS	I	128/76	0.86 [0.54;1.37]	0.51992	0.166
	II	111/76	0.68 [0.43;1.07]	0.09603	
	III	58/44	1.38 [0.76;2.52]	0.29201	
rISS	low	78/47	0.79 [0.44;1.42]	0.42723	0.974
	med	160/105	0.77 [0.53;1.14]	0.18995	
	high	30/29	0.89 [0.42;1.90]	0.75977	
Cytogenetic risk	low	194/121	0.72 [0.50;1.04]	0.07667	0.407
	high	63/48	0.98 [0.55;1.74]	0.94438	
Elevated LDH	no	249/158	0.89 [0.65;1.22]	0.48518	0.134
	yes	50/40	0.58 [0.30;1.09]	0.08947	
RI	no	278/182	0.76 [0.57;1.02]	0.06467	0.031
	yes	28/21	2.02 [0.85;4.80]	0.11064	
del17p13	no	238/155	0.76 [0.56;1.05]	0.09551	0.420
	yes	29/21	1.10 [0.47;2.60]	0.82714	
t(4;14)	no	238/153	0.82 [0.60;1.13]	0.22440	0.888
	yes	28/23	0.81 [0.35;1.84]	0.60872	
gain1q21	no	238/152	0.76 [0.55;1.05]	0.09708	0.899
	yes	20/17	0.76 [0.28;2.07]	0.58508	
t(11;14)	no	205/137	0.78 [0.56;1.10]	0.15752	0.756
	yes	60/38	0.96 [0.50;1.85]	0.89642	
Hyperdiploidy	non.HD	134/88	0.94 [0.62;1.44]	0.78731	0.373
	HD	124/84	0.68 [0.44;1.04]	0.07515	
Age	<=60	226/150	0.90 [0.65;1.24]	0.52258	0.375
	>60	80/53	0.66 [0.38;1.13]	0.13324	
All		306/203	0.83 [0.63;1.09]	0.17947	

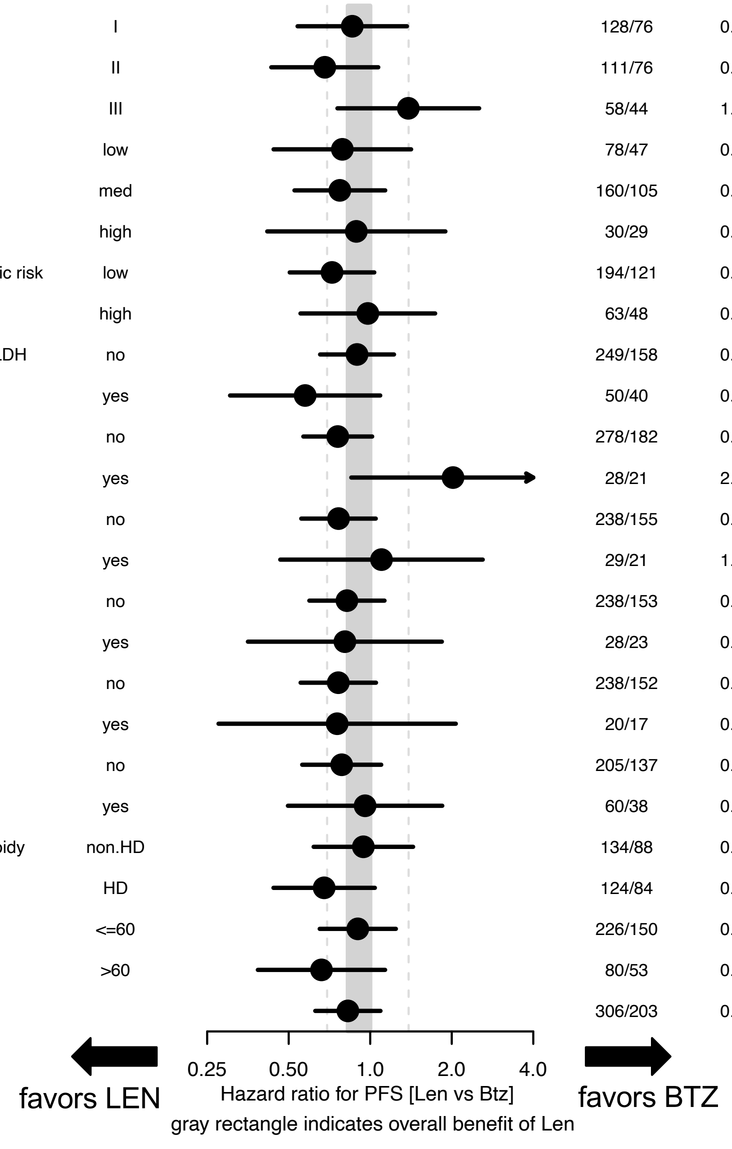

Supplemental figure 2: Univariate subgroup analyses on progression-free (PFS) according to baseline factors

Univariate subgroup analyses were calculated for PFS in the overall cohort. ISS: international staging system; rISS: revised ISS; cytorisk: cytogenetic risk status (high vs. standard [=low] risk; ldhrisk: LDH (elevated [yes] vs. normal [no]); crearisk: serum creatinine (>2 mg/dl [yes] vs. <2 mg/dl [no]); diploidy: cytogenetic hyperdiploidy [HD].

Supplemental Figure 2
