# Supplementary material for: Lenalidomide versus bortezomib maintenance after frontline autologous stem cell transplantation for multiple myeloma
Source: Blood Cancer J. 2021 Jan 7;11(1):1. doi: 10.1038/s41408-020-00390-3 (PMC7791127; doi:10.1038/s41408-020-00390-3)
Supplement: Supplementary file 4 — Supplementary Figure 3 [file 41408_2020_390_MOESM4_ESM.pdf]

subgroup		N/E	HR [95%CI]	p–value	i-p
ISS	I	134/16	0.94 [0.34; 2.57]	0.90340	0.055
	II	115/27	0.39 [0.17; 0.91]	0.02896	
	III	63/22	1.40 [0.60; 3.29]	0.43580	
rISS	low	80/7	1.17 [0.25; 5.44]	0.84386	0.357
	med	170/37	0.47 [0.24; 0.93]	0.03034	
	high	31/17	0.98 [0.37; 2.59]	0.97216	
Cytogenetic risk	low	205/34	0.39 [0.18; 0.82]	0.01315	0.020
	high	65/19	1.38 [0.54; 3.51]	0.50210	
Elevated LDH	no	260/42	0.97 [0.53; 1.80]	0.93353	0.065
	yes	54/23	0.36 [0.15; 0.85]	0.01943	
RI	no	291/58	0.56 [0.33; 0.95]	0.03269	0.009
	yes	30/9	3.15 [0.78;12.76]	0.10802	
del17p13	no	250/45	0.56 [0.30; 1.01]	0.05590	0.029
	yes	31/12	2.06 [0.61; 6.96]	0.24316	
t(4;14)	no	252/49	0.72 [0.41; 1.28]	0.26413	0.998
	yes	28/7	0.69 [0.15; 3.10]	0.63109	
gain1q21	no	250/47	0.58 [0.32; 1.05]	0.07062	0.453
	yes	21/6	1.08 [0.20; 5.92]	0.92823	
t(11;14)	no	215/47	0.81 [0.45; 1.45]	0.48096	0.329
	yes	63/9	0.39 [0.10; 1.59]	0.19127	
Hyperdiploidy	non.HD	137/35	0.90 [0.46; 1.76]	0.75371	0.266
	HD	134/21	0.57 [0.24; 1.37]	0.20813	
Age	<=60	238/44	0.85 [0.47; 1.56]	0.60689	0.238
	>60	83/23	0.43 [0.18; 0.99]	0.04773	
All		321/67	0.70 [0.43; 1.13]	0.14528	

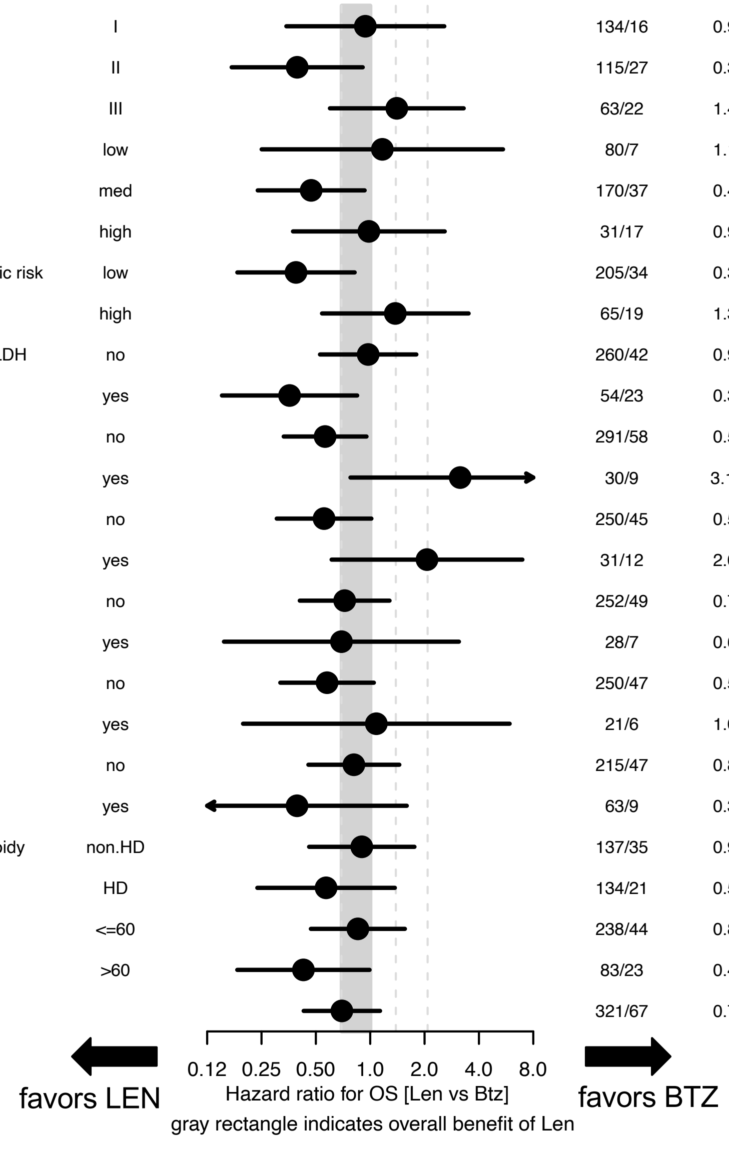

Supplemental figure 3: Subgroup analyses on overall survival (OS) according to baseline factors

Univariate subgroup analyses were calculated for OS in the overall cohort. ISS: international staging system; rISS: revised ISS; cytorisk: cytogenetic risk status (high vs. standard [=low] risk; ldhrisk: LDH (elevated [yes] vs. normal [no]); crearisk: serum creatinine (>2 mg/dl [yes] vs. <2 mg/dl [no]); diploidy: cytogenetic hyperdiploidy [HD].

Supplemental Figure 3
